# Neurocognition in stimulant addiction: commentary on Kendrick et al (2021)

**DOI:** 10.1093/psyrad/kkab008

**Published:** 2021-06-16

**Authors:** T W Robbins

**Affiliations:** Department of Psychology, Behavioural and Clinical Neuroscience Institute, University of Cambridge, UK

It would be easy to assume that that abusing stimulant drugs such as amphetamine, MDMA (‘Ecstasy’), and cocaine deleteriously impacts the brain and cognitive function. However, logically it could be the case that any neurobehavioural changes seen in drug abusers might have antedated drug taking, and so have been initially latent. An important issue is then to decide whether such changes might have been predisposing or causal factors for drug taking. They might even be considered as possible intermediate phenotypes or endophenotypes according to Gottesman's original concept (Gottesman and Goulds, 2003)—suggestive of possible constitutional or genetic factors leading to addiction. Such a hypothesis is commonly addressed by following the first degree relatives of the drug abuser to determine whether the neurobehavioural changes are also present in them. Of course, any such change might also be attributable to other familial factors, for example, the common environment, including possible stressful factors that may also contribute to neurobehavioural changes.

However, assuming that stimulant drug taking itself may also lead to brain and behavioural changes, based on evidence from animal studies where drug administration can be carefully controlled (George *et al*., 2008), and on correlations of duration or extent of drug talking with structural brain changes (Ersche *et al*. 2011a), several important questions remain: to what extent do any neurobehavioural changes produced by the drugs themselves contribute further to a drive to compulsive stimulant use (or addiction)? What is the more general nature of these changes, which might, for example, include such functions as memory and attention? To what extent would these cognitive deficits impede rehabilitation? Can they be remediated? Or do they recover over time? A further important issue is whether the type of drug abuse—for example, opioids, cannabis, and alcohol, as well as polydrug abuse including stimulants—affects the outcome.

Several studies have investigated these questions using functional neuroimaging methods generally in humans and neural interventions in animal studies, and there is support for most, if not all, of these cascading causal facets of stimulant addiction. Thus, there is evidence, for example, of impaired response inhibition processes in stimulant abusers and their siblings that correlates with a reduction of white matter in the vicinity of the right inferior frontal gyrus (Ersche *et al*., 2012). This supports a hypothesis that impulsivity may lead to addiction, presumably by promoting risky decision making. It fits with evidence in experimental animals that impulsive responding is associated with a down-regulation of dopamine D2 receptors in the nucleus accumbens, mirroring results in human cocaine abusers (Dalley *et al*., 2007). This tendency to impulsivity in rats has been shown to predict compulsive cocaine seeking, and one prominent hypothesis links this to a devolution of control from ventral to dorsal striatum, accompanied by impaired top-down control from the prefrontal cortex over habit development, known to be mediated by the dorsal striatum, specifically the putamen. Hence, compulsive drug seeking is hypothesized in part to represent a change in the balance between goal-directed behaviour and habits in favour of the latter (Everitt and Robbins, 2016). Whether this bias is also evident before drug taking or in first degree relatives is, however, more controversial. For example, some studies have observed evidence of a larger putamen in the first degree relatives of stimulant abusers (Ersche *et al*., 2013), which could be interpreted to represent a greater propensity to habit learning, if one assumes (as for the celebrated example of the hippocampus of taxi drivers, McGuire *et al*., 2000) that greater use implies greater structural change. Additionally, recent studies of resting state functional connectivity have suggested less connectivity of the ventromedial prefrontal cortex and caudate nucleus, both in stimulant abusers and in their relatives, suggestive of an impaired ‘goal-directed system’, in relation to the ‘habit system’ (Ersche *et al*., 2020).

An important limitation of many of the studies described here is that they are cross-sectional in nature; that is, they generally measure a snapshot of neurocognitive function at a particular time that is almost invariably after drug abuse has begun. Definitive evidence of causal factors in addiction should ideally include measures at several points in an individual's life history, i.e. before drug abuse, during the course of abuse, and ideally after long-term abstinence, to determine whether any neurobehavioural or cognitive effects are permanent. Largely on the basis of on cross-sectional studies, however, it is generally believed that for many individuals there are long-lasting neurocognitive deficits that diminish over time during abstinence (Ersche *et al*., 2006).

Hence, the current study by Kendrick *et al*. (2021) is of special interest as it examines brain structure and cognitive function in a prospective study of 17 very early stimulant users, repeated at 12 months. Two sub-groups of drug users, apparently matched according to demographic and premorbid indices, are identified according to subsequent LOW or HIGH stimulant usage. A comprehensive cognitive test battery reveals deficits in the HIGH group in verbal and working memory and attentional function that correlated strongly with reductions in grey matter in the putamen. This study is interesting in deflecting focus from the reductions in grey matter that have been reported to occur in the prefrontal and temporal cortex in stimulant abusers and that have been associated with cognitive impairments (Porrino *et al*., 2010). The reduction in putamen volume is not consonant with the simple notion that putamen size reflects habitual propensity to compulsive behaviour. However, it is possible, as the authors suggest, that this change may occur at a later point in stimulant addiction. The possible involvement of the putamen in cognitive deficits is intriguing in view of the considerable evidence that cognitive impairment in Parkinson's disease, including in working memory and attention, may be partly due to dopamine-dependent reductions in the dorsal striatum, including the putamen (Cheesman *et al*. 2005 ). While such cognitive deficit is usually linked to caudate nucleus involvement, a recent study has revealed correlations of cognitive and attentional impairments to reduced putamen volume (Hunerli *et al*., 2019). Moreover, planning impairments in patients with obsessive-compulsive disorder have been associated with reduced connectivity of the putamen with the dorsolateral prefrontal cortex (Vaghi *et al*., 2017). A recent publication in marmoset monkeys has shown impairments in serial reversal learning following inactivation of the putamen (Jackson *et al*., 2019), behaviour that is often impaired in stimulant abusers (Ersche *et al*., 2011b). Other recent studies have highlighted the putamen as an early focus of effects of stimulant abuse. Thus, ongoing stimulant use has been associated with reduced functional connectivity of the ventrolateral putamen, supramarginal gyrus, and insula, presumably because of the effects of stimulants on dopamine function in the striatum (Ersche *et al*., 2020). Moreover, stimulant abusers have recently been shown to exhibit reduced glutamate turnover in the putamen, correlating with their tendencies to ‘automaticity’ as measured by a habit questionnaire (Ersche *et al*., 2021). The present study therefore places a new focus on how the putamen may also contribute cognitive impairments in stimulant abusers. It will be interesting in future studies of a larger sample of these patients to track the evolution of these changes, especially following abstinence.

## References

[bib1] Cheesman AL, Barker RA, Lewis SJG et al. (2005) Lateralisation of striatal function: evidence from 18F-dopa PET in Parkinson's disease. J Neurol Neurosurg Psychiatry. 76:1204–10. 16107352 10.1136/jnnp.2004.055079 PMC1739780

[bib2] Dalley J.W, Fryer TD, Brichard L. et al. (2007) Nucleus accumbens D2/3 receptors predict trait impulsivity and cocaine reinforcement. Science. 315:1267–70. 17332411 10.1126/science.1137073 PMC1892797

[bib4] Ersche KD, Barnes A, Jones PS et al. (2011a) Abnormal structure of frontostriatal brain systems is associated with aspects of impulsivity and compulsivity in cocaine dependence. Brain. 134:2013–24. 21690575 10.1093/brain/awr138 PMC3122375

[bib3] Ersche KD, Clark L, London M et al. (2006) Profile of executive and memory function associated with amphetamine and opiate dependence. Neuropsychopharmacology. 31:1036–47. 16160707 10.1038/sj.npp.1300889 PMC1867318

[bib6] Ersche KD, Jones PS, Williams GB et al. (2012) Abnormal brain structure implicated in stimulant drug addiction. Science. 335:601–4. 22301321 10.1126/science.1214463

[bib9] Ersche KD, Lim TV, Murley A et al. (2021) Reduced glutamate turnover in the putamen is linked with automatic habits in human cocaine addiction. Biol Psychiatry. 89:970–9. 33581835 10.1016/j.biopsych.2020.12.009 PMC8083107

[bib8] Ersche KD, Meng C, Ziauddeen H et al. (2020) Brain networks underlying vulnerability and resilience to drug addiction. Proc Natl Acad Sci USA. 117:15253–61. 32541059 10.1073/pnas.2002509117 PMC7334452

[bib5] Ersche KD, Roiser JP, Abbott S et al. (2011b) Response perseveration in stimulant dependence is associated with striatal dysfunction and can be ameliorated by a D(2/3) receptor agonist. Biol Psychiatry. 70:754–62. 21967987 10.1016/j.biopsych.2011.06.033

[bib7] Ersche KD, Williams GB, Robbins TW et al. (2013) Meta-analysis of structural brain abnormalities associated with stimulant drug dependence and neuroimaging of addiction vulnerability and resilience. Curr Opin Neurobiol. 23:615–24. 23523373 10.1016/j.conb.2013.02.017

[bib10] Everitt BJ, Robbins TW (2016) Drug addiction: updating actions to habits to compulsions ten years on. Annu Rev Psychol. 67:23–50. 26253543 10.1146/annurev-psych-122414-033457

[bib11] George O, Mandyam CD, Wee S et al. (2008) Extended access to cocaine self-administration produces long-lasting prefrontal cortex-dependent working memory impairments. Neuropsychopharmacology. 33:2474–82. 18033234 10.1038/sj.npp.1301626 PMC2760333

[bib12] Gottesman IG, Gould TD (2003) The endophenotype concept in psychiatry: etymology and strategic intentions. Am J Psychiatry. 160:636–45. 12668349 10.1176/appi.ajp.160.4.636

[bib13] Hunerli D, Emek-Savas DD, Cavusoglu B et al. (2019) Mild cognitive impairment in Parkinson's disease is associated with decreased P300 amplitude and reduced putamen volume.Clin Neurophysiol. 130:1208–17. 31163365 10.1016/j.clinph.2019.04.314

[bib14] Jackson SAW, Horst NK, Axelsson SFA et al. (2019) Selective role of the putamen in serial reversal learning in the marmoset. Cereb Cortex. 29:447–60. 30395188 10.1093/cercor/bhy276 PMC6294407

[bib15] Kendrick KM, Daumann J, Koester P et al. (2021) A prospective longitudinal study shows putamen volume is associated with moderate amphetamine use and resultant cognitive impairments. Psychoradiology. 1:3–12. 10.1093/psyrad/kkab001 PMC10917237 38665308

[bib16] McGuire EA, Gadian DG, Johnsrude I et al. (2000) Navigation-related structural change in the hippocampi of taxi-drivers. Proc Natl Acad Sci USA. 97:4398–403. 10716738 10.1073/pnas.070039597 PMC18253

[bib17] Porrino LJ, Hanlon CA, Gill KE et al. (2010) Parallel studies of neural and cognitive impairment in humans and monkeys. In: Robbins TW, Everitt BJ, Nutt DJ (eds), The Neurobiology of Addiction. Oxford: Oxford University Press; 241–56.

[bib18] Vaghi MM, Vértes PE, Kitzbichler MG et al. (2017) Specific frontostriatal circuits for impaired cognitive flexibility and goal-directed planning in obsessive-compulsive disorder: evidence from resting-state functional connectivity. Biol Psychiatry. 81:708–17. 27769568 10.1016/j.biopsych.2016.08.009 PMC6020061

